# Treatment of shoulder sequelae in brachial plexus birth injury

**DOI:** 10.3109/17453674.2011.588855

**Published:** 2011-09-02

**Authors:** Tiina Pöyhiä, Antti Lamminen, Jari Peltonen, Patrick Willamo, Yrjänä Nietosvaara

**Affiliations:** ^1^Helsinki Medical Imaging Center; ^2^Department of Surgery, Hospital for Children and Adolescents; ^3^Physiotherapy, Hospital for Children and Adolescents, Helsinki University Central Hospital, Helsinki, Finland; Correspondence: tiina.poyhia@hus.fi

## Abstract

**Background:**

Many children with permanent brachial plexus birth injury (BPBI) develop shoulder problems, with subsequent joint deformity without treatment. We assessed the indications and outcome of shoulder operations for BPBI.

**Patients and methods:**

31 BPBI patients who had undergone a shoulder operation in our hospital between March 2002 and December 2005 were included in the study. Relocation of the humeral head had been performed in 13 patients, external rotation osteotomy of the humerus in 5 patients, subscapular tendon lengthening in 5 patients, and teres major transposition in 8 patients. Subjective results were registered. Shoulder range of motion was measured, and function assessed according to the Mallet scale. Magnetic resonance imaging (MRI) was performed pre- and postoperatively. Glenoscapular angle (GSA) and percentage of humeral head anterior to the middle of the glenoid fossa (PHHA) were measured. Congruency of the glenohumeral joint (GHJ) was estimated. The mean follow-up time was 3.8 (1.7–6.8) years.

**Results:**

At follow-up, the subjective result was satisfactory in 30 of the 31 patients. There were 4 failures, which in retrospect were due to wrong choice of surgical method in 3 of these 4 patients. Mean increase in Mallet score was 5.5 after successful relocation, 1.4 after rotation osteotomy, 2.2 after subscapular tendon lengthening, and 3.1 after teres major transposition. Congruency of the shoulder joint improved in 10 of 13 patients who had undergone a relocation operation, with mean improvement in GSA of 33º and mean increase in PHHA of 25%. There were no substantial changes in congruency of the glenohumeral joint in patients treated with other operation types.

**Interpretation:**

Restriction of the range of motion and malposition of the glenohumeral joint can be improved surgically in brachial plexus birth injury. Remodeling of the joint takes place after successful relocation of the humeral head in young patients.

The incidence of brachial plexus birth injury (BPBI) has been calculated to be 3.1–3.8 per 1,000 live births, and the incidence of BPBI-related posterior subluxation of the humeral head 0.21–0.28 per 1,000 live births in recent population-based Scandinavian studies (Dahlin et al. 2007, Pöyhiä et al. 2010). BPBI proceeds craniocaudally in babies born by vertex presentation (99% of births in Finland according to National Birth Register data); upper root injury appears in practically all of these BPBI cases. Injured nerve roots C5-C6 result in weakness of the external rotators, while internal rotators dominate in most patients with BPBI. This muscle imbalance of the affected shoulder can lead to posterior subluxation of the humeral head, with subsequent deformity of the glenohumeral joint (GHJ), leading to restricted external rotation of the arm and diminished function.

In recent studies, posterior subluxation of the humeral head has been detected with ultrasound as early as at 3 months of age (Moukoko et al. 2004, Pöyhiä et al. 2010). Routine follow-up of patients with permanent BPBI has been advocated to prevent progression of posterior subluxation of the humeral head to joint deformities.

MRI is of value for assessment of the degree of glenoid retroversion and possible posterior subluxation of the humeral head, as well as the condition of the muscles in children who have already developed internal rotation contracture or incongruency of the GHJ (Pöyhiä et al. 2005). This information is important in selecting the optimal surgical technique for each patient. Rebalancing soft tissue operations with tendon lengthening and tendon transfers may be sufficient in patients with minor changes of the GHJ. Muscle imbalance and shoulder deformities have been treated with tendon lengthening and muscle transfers (Gilbert et al. 1988, 1991, Narakas 1993, Phipps and Hoffer 1995, Nath and Paizi 2007, Bertelli 2009). Other authors believe that relocation of the humeral head to improve the function of the shoulder is the most important surgical intervention in young patients with incongruent shoulders (van Sluijs et al. 2004, Kambhampati et al. 2006). External rotation osteotomy is recommended in advanced glenohumeral deformity with pseudoglenoid and posterior dislocation of the humeral head in older patients (Goddard and Fixsen 1984, Kirkos and Papadopoulos 1988, Al-Qattan 2002, Waters and Bae 2006, Bae and Waters 2007).

In this prospective study we assessed the indication settings, functional results, and glenohumeral congruity after surgical treatment of shoulder impairment in BPBI.

## Patients and methods

34 patients with permanent BPBI (20 boys) and impaired shoulder function underwent surgery between March 2002 and December 2005 at the Hospital for Children and Adolescents, Helsinki University Central Hospital. 2 of the patients failed to attend a preoperative MRI study and were excluded from the study. A third patient was excluded because the operation performed was not in accordance with the study design. The extent of the BPBI in the remaining 31 patients was as follows: upper roots (C5-C6), 7 patients; upper and middle roots (C5-C7), 15 patients; and total injury (C5-Th1), 9 patients. Plexus reconstruction had been performed on 9 patients before shoulder surgery (Table 1).

**Table 1.** Patient demographics with pre- and postoperative measurements

A	B	C	D	E		F		G		H		I		J		K
1	2.7	total	10	-60	-15	4	38	4	2	p	c	0	10	90	45	R
2	0.9	C5-7		–60	–15	5	38	4	2	p	c	–20	50	90	80	R **^a^**
3	4	C5-6		–55	–6	7	40	4	2	p	c	–15	20	70	70	R **^b^**
4	4.7	C5-7		–50	–2	7	44	4	2	p	c	–20	45	120	75	R **^a^**
5	3	C5-7		–47	–47	5	5	4	4	p	p	0	–20	145	180	R
6	7.7	C5-7		–47	–47	17	15	4	4	p	p	–20	–20	180	180	R
7	3.5	C5-6		–40	–20	15	38	4	2	p	c	–20	0	110	160	R **^a^**
8	2.3	C5-7		–40	–10	15	40	4	2	s	c	–45	25	140	180	R
9	1.8	C5-7		–35	–1	28	40	4	2	s	c	0	40	130	110	R
10	4	C5-7	6	–30	–2	23	44	3	1	p	c	–20	80	90	130	R
11	6.9	total		–25	–25	40	40	4	4	s	s	–20	0	180	180	R
12	4.7	total	3	–24	–4	17	47	4	2	p	c	–20	0	100	130	R
13	4.6	C5-7	4	–20	–10	31	36	3	2	s	s	–20	–30	110	180	R
14	14	total		–60	–60	–1	–1	4	4	p	p	–10	15	95	120	O
15	5.7	C5-7		–50	–50	–10	–10	4	4	p	p	–20	20	100	100	O
16	7.8	C5-6		–45	–45	14	10	4	4	p	p	–20	0	120	110	O
17	8.3	C5-7		–38	–38	18	34	4	4	p	s	–30	0	160	170	O
18	12	total		–35	–35	21	21	2	2	s	s	–20	0	110	95	O
19	5.3	C5-7		–27	–27	20	10	4	4	p	p	–10	–20	140	180	ST
20	15	total	5	–15	–15	40	40	2	2	c	c	–20	0	160	180	ST
21	11	C5-7		–9	–2	36	45	1	1	c	c	–20	15	160	180	ST
22	5.7	C5-7		–7	–7	48	48	1	1	c	c	–20	0	170	170	ST
23	13	C5-6		–7	–3	38	45	1	1	c	c	–10	10	160	160	ST
24	5.5	C5-7		–21	–10	37	47	2	2	c	c	–20	0	150	170	TM **^c^**
25	3.8	C5-7		–19	–5	48	48	2	2	c	c	0	45	90	100	TM **^c^**
26	5.2	total	3	–14	–14	37	37	1	1	c	c	0	0	90	180	TM
27	4.4	C5-6	5	–10	–5	46	50	2	2	c	c	0	0	90	160	TM
28	7.4	C5-6	5	–7	–7	43	43	1	1	c	c	0	–20	180	170	TM
29	4.6	total	5	–7	–7	48	46	1	1	c	c	–30	10	90	120	TM
30	11	total		–5	–1	45	45	1	1	c	c	–20	20	160	180	TM **^c^**
31	8.6	C5-6		–3	–3	54	53	1	1	c	c	0	10	120	100	TM

A Case no. B Age at surgery C Roots injured D Age (in months) at the time of plexus reconstruction E Glenoscapular angle (GSA) (°) (preop. and postop.) F Percentage of humeral head anterior to the middle of glenoid fossa (PHHA) (%) (preop. and postop.) G Type of glenoid (1 = concentric, 2 = flat, 3 = biconcave, and 4 = pseudoglenoid) (preop. and postop.) H Congruency of the glenohumeral joint (preop. and postop.) c = congruent s = subluxation of the humeral head p = severely deformed, pseudoglenoid I Active external rotation in adduction (°) (preop. and postop.) J Elevation (°) (preop. and postop.) K Type of operation R = relocation O = osteotomy ST = subscapular tendon lengthening TM = teres major transposition

**^a^** additional teres major transposition operation later.

**^b^** additional osteotomy operation later.

**^c^** subscapular lengthening later.

**Figure 1.**
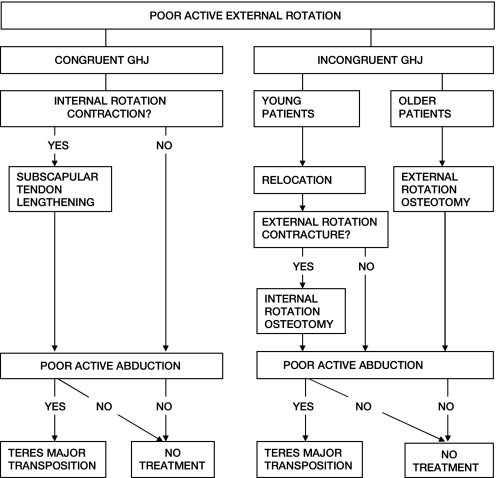
Treatment flow chart for brachial plexus birth injury with poor active external rotation. GHJ = glenohumeral joint.

The primary goal of the treatment was to restore the glenohumeral joint congruency in young patients. The secondary goal of the surgery was to improve motion of the shoulder. The tertiary indication for treatment was to optimize the sector of movement of shoulder rotation in older patients with deformed shoulder joint. The most important denominator in selection of the operation method was congruency of the GHJ, estimated with clinical examination and measurements of glenoscapular angle (GSA) and percentage of humeral head anterior to the middle of the glenoid fossa (PHHA). The treatment flow chart is shown in Figure 1.

Young patients (aged 0.9–7.7 years, n = 13) with posterior shoulder subluxation and deformity (median passive external rotation preoperatively 0º (range –20 to 45, SE 6.6)) were treated by relocation (GSA = –60 to –20º, PHHA = 4–40%) of the humeral head through a deltopectoral incision. 11 of the patients had a pseudoglenoid, and the remaining 2 had a biconcave glenoid. In addition to subscapular tendon lengthening and discision of the coracohumeral ligament, the coracoid process was shortened in 10 of these 13 patients. 4 of the 10 patients with a successful relocation had an additional operation later: a teres major transposition to infraspinous muscle was performed in 3 patients and an internal rotation osteotomy of the humerus in 1 patient.

External rotation osteotomy of the humerus was performed above the deltoid tuberosity for older patients (age 5.7–14 years, n = 5) with preoperative median passive external rotation of –20º (range –30 to 0) and with a deformed GHJ (GSA = –60 to –35º, PHHA = –10 to 21%). The distal humerus was externally rotated 30– 40º, and the osteosynthesis was performed with a plate.

5 patients with internal rotation contracture (passive external rotation between –20º and 0º and good active elevation (140 –170º)), but without major shoulder joint deformity, were treated by subscapular tendon lengthening through a deltopectoral incision.

8 patients with poor active elevation and/or poor external rotation in abduction without major shoulder joint deformity (limited active elevation (range 90–180º) and active external rotation in abduction median 17.5º (range 0–45, SE 5.6) were treated by teres major to infraspinous transposition. Subscapular tendon lengthening was performed in 3 of these patients with concomitant internal rotation contracture.

Postoperative immobilization with a thoracobrachial cast was used for mean 5 (3–7) weeks after relocation, 3–4 weeks after subscapular lengthening, and 4–5 weeks after teres major transposition, and with a collar and cuff for 3–4 weeks after humeral osteotomy.

The opinion of the patient (aged > 7 years) or the parents (children under 7 years of age) was recorded at the time of final follow-up using a 3-scale system: “worse than preoperatively”, “no benefit”, or “better than before the operation”. Possible complications were registered. External rotation in adduction and abduction as well as elevation of the arm were measured with a goniometer by a physiotherapist (PW), both preoperatively and postoperatively. Functional assessment of the shoulder was performed using the Mallet classification (Mallet 1972). For MRI, the mean follow-up time was 3.8 (1.7–6.8) years.

### Imaging

The affected shoulder of all patients was imaged with MRI both pre- and postoperatively. Imaging studies were done with a 1.5 T MRI unit (Siemens, Erlangen, Germany, or Philips Medical Systems, Achieva, Best, the Netherlands) with a surface coil. During the MR examination, the patient was in a supine position on the table with the arm alongside the body. The following sequences were obtained (Siemens): T1-weighted spin echo images in axial, oblique coronal, and oblique sagittal planes (TR 817 ms, TE 20 ms, TA 6 min 2 s, matrix 220 × 256, FoV 160 × 138 mm) and T2*-weighted 2D gradient echo images in the axial plane (TR 944.3 ms, TE 25.8 ms, flip angle 30º, TA 4 min 2 s, matrix 256 × 256, FoV 220 × 220 mm). The corresponding parameters for the Philips MRI unit were T1-weighted spin echo images in axial, oblique coronal, and oblique sagittal planes (TR 500 ms, TE 15 ms, TA 4 min, matrix 240 × 240, FoV 160 × 144 mm) and 3D WATSc sequence in the axial plane (TR 20 ms, TE 7.7 ms, flip angle 25º, TA 5 min 47 s, matrix 288 × 232, FoV 160 × 129 mm). Slice thickness was 2 mm in the Philips 3D WATSc sequence and 4.0 mm in all other sequences. The measurements were performed using the IMPAX for Orthopedics software tool (Agfa-Gevaert Group, Mortsel, Belgium).

**Figure 2.**
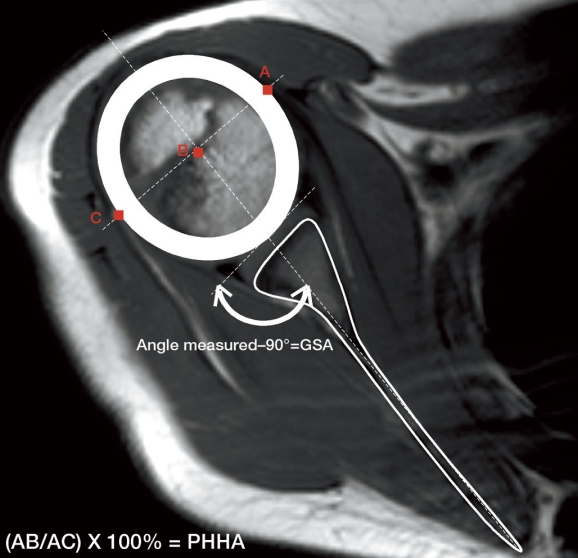
Schematic image showing the measurement of glenoscapular angle (GSA) and the percentage of humeral head anterior to the middle of the glenoid fossa (PHHA): i.e. (AB/AC) × 100%.

**Figure 3.**
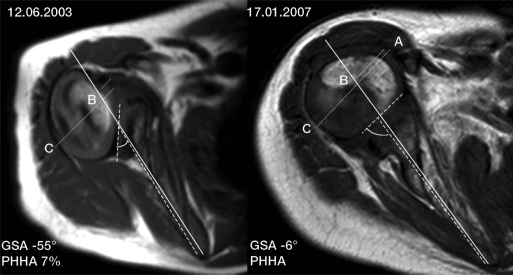
Axial T1-W images of the right glenohumeral joint of a boy with BPBI-related restricted external rotation. Relocation operation performed at the age of 4 years. a) Preoperatively: pseudoglenoid with severe retroversion of the right glenoid fossa, GSA = -55°; PHHA = 7% at the age of 3 years and 4 months. b) Postoperatively: glenohumeral joint with GSA = -6° and PHHA = 40% at the age of 7 years.

Glenoscapular angle (GSA) was measured by drawing 2 lines according to Friedman et al. (1992). The first line was drawn between the anterior and posterior corners of the glenoid cartilage. A bisecting line was drawn from the medial tip of the scapula to the midpoint of the glenoid. The angle between the first line and the line along the axis of the scapula was measured. GSA was obtained by subtracting 90° from the posteromedial quadrant angle (Figure 2). The more negative the GSA value, the worse the retroversion of the glenoid. In the case of the pseudoglenoid, the posteriorly retroverted glenoid was measured as the angle of version (Figure 3). Percentage of humeral head anterior to the middle of the glenoid fossa (PHHA), measured according to Waters et al. (1998), revealed the percentage of the humeral head that was located anterior to the line along the long axis of the scapula going through the midpoint of the glenoid surface (Figure 2). Normally, approximately half of the humeral head is located anterior to the line that runs along long axis of the scapula, passing through the midpoint of the glenoid surface. In this normal situation, the PHHA value is 50%. The worse the posterior subluxation, the lower the PHHA value. The shape of the glenoid was classified according to Pearl et al. (2003) as being either normal concentric, flat, biconcave, or pseudoglenoid. GHJ was classified as congruent, subluxated, or severely deformed, pseudoglenoid with the humeral head articulating with the deformed posteriorly retroverted glenoid, which is in a different plane from the normal glenoid.

### Statistics

Values of ROM are shown as median (range) with SE values. Data of summed Mallet scores were expressed with mean values and standard deviations. Wilcoxon test has been used to compare pre- and postoperative Mallet and GSA values in different operation groups. Any value of p < 0.05 was considered significant.

The study was approved by the ethics committee of the hospital on March 2, 2001 (Dnr: 79/E7/2001) and informed consent was obtained from the guardians of all children.

## Results

Patient characteristics and outcome are presented in Table 1. One patient in the relocation group had a deep wound infection requiring revision surgery. No other complications occurred. After subscapular tendon lengthening, 1 patient did not experience any improvement from the operation. All the other patients or parents felt that they had benefited from the operation.

**Figure 4.**
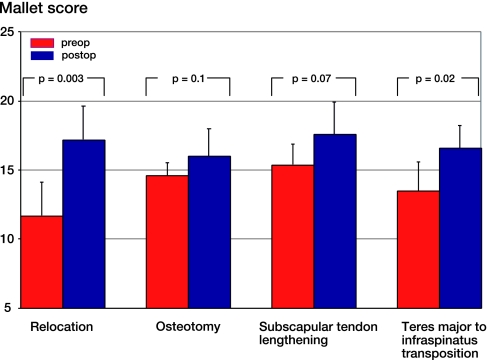
Mean values and standard deviations of summed Mallet scores in different types of operations. Exact p-values are given for statistical comparisons of pre-and postoperative measurements.

13 patients underwent relocation of the humeral head. 2 patients over 6 years of age and 1 patient with immobilization time that was too short (4 weeks) developed resubluxation of the humeral head, and these were considered failed procedures without major functional or radiological improvement. After successful relocation (10/13), the median value of passive external rotation postoperatively was 48º (range10–80, SE 8). Active external rotation improved in 10 patients in adduction (median increase 38° (range 10–100, SE 9)) and in abduction (median 48° (range 15–80), and elevation improved in 6 patients postoperatively (median 40° (range 30–70)). Mean increase in summed Mallet score was 5.5 (SD 3.0) after the successful relocation operations (Table 2 and Figure 4). All patient-specific Mallet score values pre-and postoperatively are given in Table 3. In 10 patients, mean improvement in postoperative GSA was 33° (SD 14) and mean improvement in postoperative PHHA was 25% (SD 10). The difference between pre- and postoperative GSA values in the relocation group was statistically significant (p = 0.005). The shape of the glenoid improved in 10 patients postoperatively (9 flat, 1 concentric) (Figure 3).

**Table 2.** Mean Mallet scores pre- and postoperatively

	Abduction		External rotation		Hand to neck		Hand to back		Hand to mouth	
	Preop. Postop.		Preop. Postop.		Preop. Postop.		Preop. Postop.		Preop. Postop.	
Relocation	3.6	3.8	1.2	3.2 **^a^**	2.4	3.7 **^a^**	2.7	3.0	2.1	3.5 **^a^**
Osteotomy	4.0	4	1.6	2.4 **^a^**	2.6	3.4	3.8	2.6	2.6	3.6
ST	4.2	4.8	1.0	2.2	3.2	4.0 **^a^**	3.8	3.4	3.2	3.2
TM	3.4	4.3	1.6	2.5	2.5	3.7 **^a^**	3.1	2.9	2.9	3.2

ST: subscapular tendom lengthening; TM: teres major to infraspinatus transposition.

**^a^** significant difference between pre- and postoperative values (Wilcoxon).

**Table 3.** All patient-specific Mallet score values pre- and postoperatively

A	B	C	D		E		F		G		H		I
1	2.7	total	3	3	2	3	2	2	2	2	1	4	R
2	0.9	C5-7	3	3	1	4	2	4	2	3	1	4	R **^a^**
3	4	C5-6	3	3	1	4	2	4	3	2	3	4	R **^b^**
4	4.7	C5-7	4	3	1	4	2	4	2	3	2	4	R **^a^**
5	3	C5-7	4	5	2	1	3	4	3	4	2	4	R
6	7.7	C5-7	5	5	1	1	4	4	4	3	4	3	R
7	3.5	C5-6	4	4	1	2	2	4	3	4	2	3	R **^a^**
8	2.3	C5-7	4	5	1	4	3	4	4	4	3	4	R
9	1.8	C5-7	4	4	2	4	3	4	3	2	1	4	R
10	4	C5-7	3	4	1	4	3	4	2	4	2	4	R
11	6.9	total	5	5	1	2	4	5	4	4	4	4	R
12	4.7	total	4	4	1	2	2	3	2	2	2	2	R
13	4.6	C5-7	4	5	1	1	3	4	3	4	4	2	R
14	14	total	4	4	2	3	3	4	4	2	2	4	O
15	5.7	C5-7	4	4	2	3	2	2	4	2	2	2	O
16	7.8	C5-6	4	4	1	2	2	4	4	3	3	4	O
17	8.3	C5-7	4	4	1	2	3	4	4	4	4	4	O
18	12	total	4	4	2	2	3	3	3	2	2	4	O
19	5.3	C5-7	4	5	1	1	3	4	4	3	3	2	ST
20	15	total	4	5	1	2	3	4	3	2	2	2	ST
21	11	C5-7	4	5	1	3	3	4	4	4	4	4	ST
22	5.7	C5-7	5	5	1	2	3	4	4	4	3	4	ST
23	13	C5-6	4	4	1	3	4	4	4	4	4	4	ST
24	5.5	C5-7	4	4	1	2	3	4	4	3	3	4	TM **^c^**
25	3.8	C5-7	3	4	2	4	2	4	3	2	3	4	TM **^c^**
26	5.2	total	3	5	2	2	2	4	2	2	2	2	TM
27	4.4	C5-6	3	4	2	2	2	4	2	3	2	2	TM
28	7.4	C5-6	5	4	2	1	3	4	4	4	3	2	TM
29	4.6	total	3	4	1	3	2	3	2	2	4	4	TM
30	11	total	4	5	1	3	3	4	4	3	2	4	TM **^c^**
31	8.6	C5-6	2	4	2	3	3	3	4	4	4	4	TM

A Case no. B Age at surgery C Roots injured D Abduction (preop. and postop.) E External rotation (preop. and postop.) F Hand to neck (preop. and postop.) G Hand to back (preop. and postop.) H Hand to mouth (preop. and postop.) I Type of operations (See Table 1)

5 patients had humeral osteotomy at a mean age of 9.4 (SD 3.2) years. Median passive external rotation postoperatively was 30º (range 0–60, SE 10). The median increase in active external rotation in adduction was 25° (range 20–40, SE 3.7) and in abduction it was 30° (range 25–45). Elevation improved in 2 of these patients. No change in GSA values or in the shape of the glenoid occurred after humeral osteotomy, but one patient had an increased PHHA of 16%.

5 patients underwent a subscapular tendon lengthening operation. Median passive external rotation postoperatively was 45º (range –20 to 80, SE 17). 4 of these patients had better active external rotation in adduction (median increase 20° (range 20–35, SE 3.7)) and in abduction (median 55° (range 45–75, SE 6.6)) postoperatively; 3 had improved elevation (median 20° (range 20–40, SE 6.6)). One of these patients (patient number 19; see Table 1) should have been treated with osteotomy. Subscapular tendon lengthening did not improve GSA, PHHA, or shape of the glenoid.

8 patients had a teres major to infraspinatus transposition, 3 of whom also had subscapular tendon lengthening. Median active external rotation in abduction was 73º (range 45–80, SE 6.0). 5 of these 8 patients showed improved active external rotation in adduction (median 40° (range 10–45, SE 6.8)), whereas elevation (median 25° (range 10–90, SE13)) improved in 6 patients. GSA, PHHA, and shape of the glenoid did not change after teres major transposition.

## Discussion

The aim of treatment of shoulder sequelae in BPBI is to maintain or restore a congruent GHJ. The final outcome after relocation of the humeral head is probably dependent on the remodeling capacity of the growth area. El Gammal et al. (2006) suggested that glenohumeral remodeling capacity decreases after 4 years of age. This is analogous to the correction of acetabular dysplasia by remodeling after treatment of congenital dislocation of the hip; after 4 years of age, this remodeling capacity is diminished (Kasser et al. 1985, Lalonde et al. 2002). Our study supports this hypothesis: 2 of our patients who failed to preserve the relocation and developed resubluxation without glenohumeral remodeling were 6.9 and 7.7 years old at the time of operation. In retrospect, these patients should have been treated with osteotomy. Our third patient with resubluxation had an immobilization time that was too short.

In internal rotation contracture of the shoulder without joint deformity, soft-tissue procedures should be sufficient to improve external rotation (Jellicoe and Parsons 2008). Tendon transfers have been proposed to stop the development of shoulder deformity: Waters et al. (2005) found little improvement in glenoid version and glenohumeral congruency after latissimus dorsi and teres major tendon transfers to the rotator cuff combined with appropriate extra-articular musculotendinous lengthenings. Minor changes in glenoid version in patients who have undergone corrective soft-tissue procedures may be due to normal growth, and glenoid version may diminish by about 6° in childhood during the first 10 years according to the study by Mintzer et al. (1996). Kozin et al. (2006) evaluated the outcome in 23 children after latissimus dorsi and teres major tendon transfers—clinically and with MRI—in a 1-year follow-up study. Functional outcome improved without positive changes in glenoid version or congruency of the GHJ. Our results confirm these findings; retroversion of the glenoid improved only after relocation of the incongruent GHJ.

Clinical assessment of whether the reduced humeroscapular external rotation is a result of muscle contracture or GHJ incongruence is not always easy. Kon et al. (2004) concluded that consistent patterns of deformity of the GHJ in BPBI verified by intraoperative arthrography correlated with internal rotation contracture. They presumed that assessment of glenoid deformity by arthrography or MRI will help in surgical planning. Torode and Donnan (1998) proposed that all children with BPBI and restricted external rotation should undergo CT scanning to detect potential posterior dislocation. We agree with the need for preoperative investigation in planning of an optimal surgical strategy, but recommend MRI instead of CT. The advantage of CT scanning is the shorter imaging time, which may allow use of the scanning procedure for cooperative children without anesthesia. However, young children may still need sedation because it is unacceptable to rescan due to motion artifacts. Furthermore, soft tissues are not properly visualized with CT and bony outlines give only indirect information about joint congruity, especially in small children. Thus, MR is a superior technique in imaging BPBP patients with shoulder sequelae since it is important to visualize the cartilaginous joint surfaces and to evaluate muscle pathology for preoperative planning.

The mean follow-up time of 3.8 years in our study is relatively short. Pagnotta et al. (2004) have expressed concern about the loss of clinical improvement, especially abduction in longer follow- up periods after latissimus dorsi transfer. Loss of initially achieved active external rotation by anterior release and teres major and latissimus dorsi transfer was reported by Kirkos et al. (2005) after a mean follow-up time of 30 years.

Various BPBI-related shoulder sequelae can be improved surgically. The choice of operation depends on GHJ congruency, presence of contractures, strength of the shoulder muscles, and the age of the patient. Deformity of the shoulder can be successfully reversed with relocation of the humeral head in patients who are younger than 5 years.
